# First person – Paola Lepanto and Florencia Levin-Ferreyra

**DOI:** 10.1242/bio.059008

**Published:** 2021-09-17

## Abstract

First Person is a series of interviews with the first authors of a selection of papers published in Biology Open, helping early-career researchers promote themselves alongside their papers. Paola Lepanto and Florencia Levin-Ferreyra are co-first authors on ‘ Insights into *in vivo* adipocyte differentiation through cell-specific labeling in zebrafish’, published in BiO. Paola is a research assistant in the lab of José Badano at Institut Pasteur Montevideo, investigating cellular and developmental biology. Florencia conducted the research described in this article while a final dissertation student in José Badano's lab at Institut Pasteur Montevideo. She is now a research associate in the lab of Bruno di Stefano at Baylor College of Medicine, Houston, USA, investigating stem cell potency and cell fate decisions.


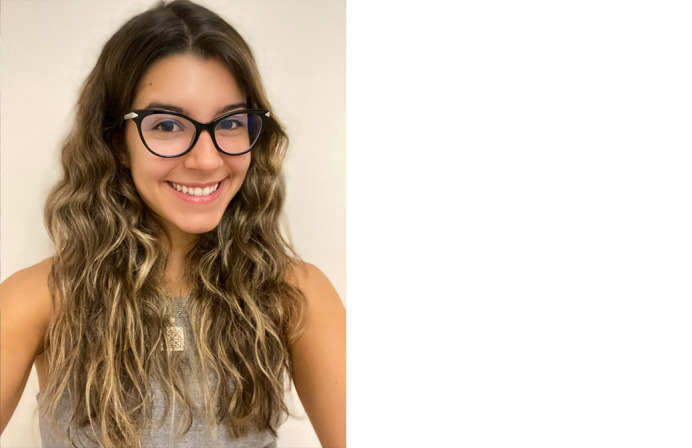


**Paola Lepanto and Florencia Levin-Ferreyra**

**What is your scientific background and the general focus of your lab?**

P.L.: Throughout my academic trajectory I′ve worked in different topics and research systems studying different aspects of cell biology, including axonal maintenance in the peripheral nervous system, bacterial-epithelial interaction in culture and retinal ganglion cell development. During the postdoc my aim was to introduce the use of zebrafish model into our lab to study adipocyte differentiation in ciliopathies, one of our main research topics, trying to offer an alternative experimental system to complement the work being carried out in murine and cultured cell models.

F.L.-F.: From the beginning of my scientific career, I had a strong predilection towards the biomedical field. During my undergraduate studies, I joined the Human Molecular Genetics laboratory where I worked with Paola on the establishment of a new zebrafish transgenic reporter line specific for adipose tissue. Feeling encouraged by the fascinating aspects of cell biology, I decided to turn my life around, so I moved out to the United States to start studying the mechanisms governing cell potency and cell fate decision, a field in which I am planning to focus on during the coming years.

**How would you explain the main findings of your paper to non-scientific family and friends?**

In this work we implemented a strategy to specifically label the boundary (plasma membrane) of adipocytes, the cell type that stores fatty acids in adipose tissue, in live zebrafish larvae. Using this approach combined with specific dyes and advanced microscopy techniques, we observed adipocytes during the accumulation of fat within the live tissue. We also discovered that during these stages adipocytes may generate membrane extensions that reach blood vessels. Furthermore, we showed that combining our cell-type specific labelling strategy, a general lipid staining method and advanced microscopy techniques it is possible to analyse the accumulation of neutral lipids in developing adipocytes within their natural context.

**What are the potential implications of these results for your field of research?**

The observation of adipocyte protrusions contacting blood vessels along differentiation highlights the importance of *in vivo* studies and will probably lead to new questions regarding the function of these interactions and their relevance in adipocyte physiology. Furthermore, we think that our work extends the tools available to study adipose tissue formation and its dynamics in live zebrafish. We hope that the combination of tools and techniques presented in our article would be of great use to gain new insights into different problems involving adipose tissue but also in other fields.

“…we think that our work extends the tools available to study adipose tissue formation and its dynamics in live zebrafish.”

**What has surprised you the most while conducting your research?**

What surprised us the most was observing the variable shapes and movement of early adipocytes *in vivo*. We are accustomed to think about these cells as bags full of fat, and sometimes we disregard the environment and interactions that these cells encounter during their differentiation. This emphasizes the importance of studying the dynamics of biology *in vivo*, not to mention that this process may be relevant in shaping the form and physiology of the mature tissue.

**Figure:**
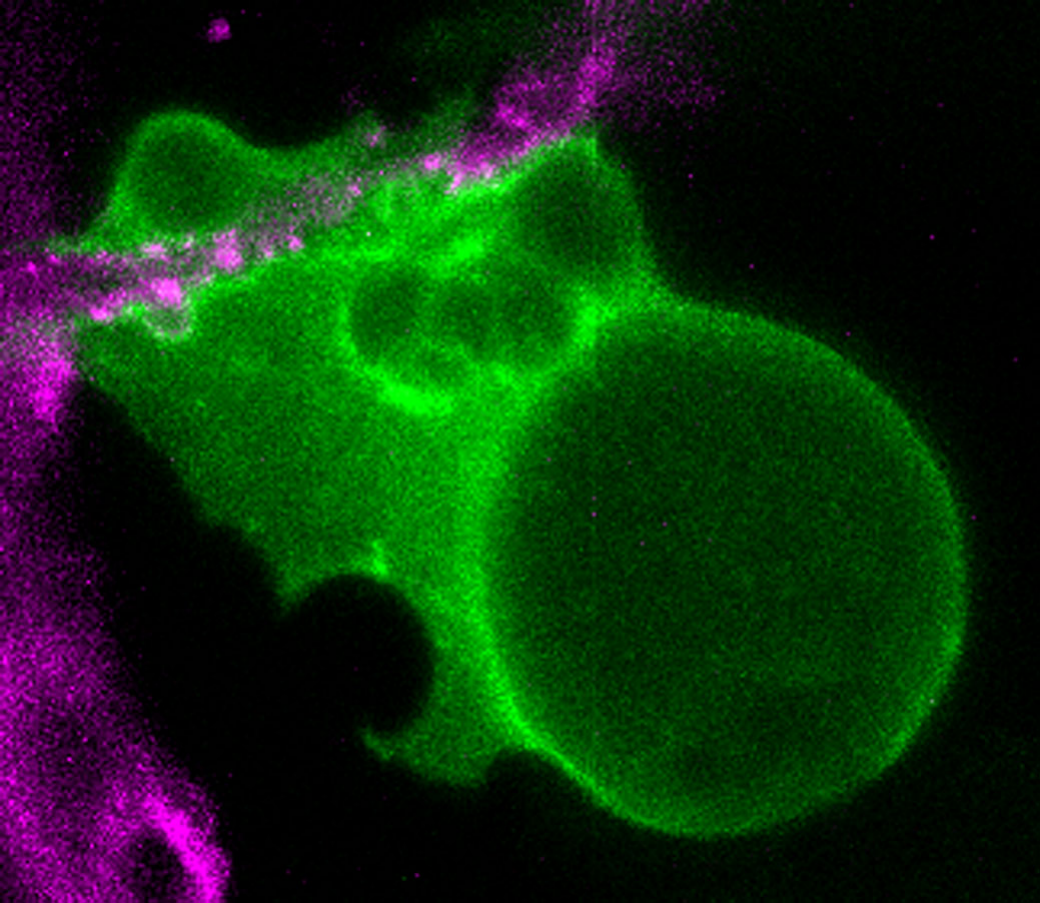
Image of a labelled adipocyte (green) interacting with a blood vessel (magenta) in a live zebrafish larva.

**What, in your opinion, are some of the greatest achievements in your field and how has this influenced your research?**

The widespread use of zebrafish led to the generation of many tools that are currently available in the community to work with this organism. These tools led to huge progress and advances that have been made in advanced microscopy techniques, and inspired us to get into this area of research, with an aim to contribute to the assessment of the cell biology of adipocytes from the *in vivo* setting.

“I almost feel that ‘making science’ is being pushed to be ‘selling science’…”

**What changes do you think could improve the professional lives of early-career scientists?**

F.L.-F.: The first obstacle that comes to my mind regarding a young scientist's career is the lack of funding opportunities together with the high level of competition that their stringent requirements also create. Applying for grants has become a stressful and time-consuming duty for most researchers, not to mention that a great portion of the opportunities are not about fundamental research but are about translational science. I almost feel that ‘making science’ is being pushed to be ‘selling science’, and is not the essence of the science we make. Moreover, I am experiencing this personally as a foreign student, since I am not able to apply to almost any grant available in the United States, representing a barrier for young scientists who want to pursue careers outside independently. Overall, I believe that increasing and diversifying funding sources will have a strong impact on the life of these researchers.

P.L.: I agree with Florencia about the need for increasing and diversifying funding to promote an increasing number of young scientists. A second issue that would improve our lives would be having career advice and planning from the early stages. Also, I think that interaction with other scientists is something that positively impacts on early-career professionals, and thus an effort should be made in encouraging interaction among colleagues and with senior researchers at every step.

**What's next for you?**

P.L.: I will continue working in a related area, mainly in the study of how ciliopathy associated genes work to affect the generation of new adipocytes *in vivo*.

F.L.-F.: In my case, I decided to pursue my PhD studies abroad. I am currently in the United States as a member of the Bruno di Stefano Lab at Baylor College of Medicine, where we mainly study the mechanisms governing cell potency and cell fate decision.

## References

[BIO059008C1] Lepanto, P., Levin-Ferreyra, F., Koziol, U., Malacrida, L. and Badano, J. L. (2021). Insights into *in vivo* adipocyte differentiation through cell-specific labeling in zebrafish. *Biol. Open* 10, bio058734. 10.1242/bio.058734 34409430 PMC8443861

